# Liquid Metal Electrocatalyst with Ultralow Pt Loading for Ethanol Oxidation

**DOI:** 10.1002/smsc.202400370

**Published:** 2024-10-06

**Authors:** Muhammad Hamza Nazir, Tu C. Le, Imtisal Zahid, Karma Zuraiqi, Mew P. Aukarasereenont, Caiden J. Parker, Pierre H. A. Vaillant, Fahad Jabbar, Chung Kim Nguyen, Mehmood Irfan, Mariam Ameen, Michelle J. S. Spencer, Andrew J. Christofferson, Salvy P. Russo, Ken Chiang, Nastaran Meftahi, Torben Daeneke, Dan Yang

**Affiliations:** ^1^ Department of Chemical and Environmental Engineering School of Engineering RMIT University Melbourne VIC 3001 Australia; ^2^ Department of Manufacturing Materials and Mechatronics School of Engineering RMIT University Melbourne VIC 3001 Australia; ^3^ School of Science RMIT University Melbourne VIC 3001 Australia

**Keywords:** direct ethanol fuel cells, electrolyte optimizations, ethanol oxidations, liquid metal catalysts, machine learning

## Abstract

Developing efficient and durable electrocatalysts for ethanol electro‐oxidation is crucial for enabling the application of direct ethanol fuel cell technology. Herein, it is demonstrated that Pt–Ga liquid metal‐based nanodroplets can serve as an efficient electrocatalyst to drive ethanol oxidation. The mass activity of Pt is significantly improved by alloying with liquid gallium. Guided by machine learning neural networks, a low‐concentration alkaline electrolyte is specifically formulated to allow electrodes with ultralow Pt loading to demonstrate remarkable activity toward ethanol oxidation with a mass activity as high as 13.47 A mg^−1^_Pt_, which is more than 14 times higher than that of commercial Pt/C electrocatalysts (i.e., 0.76 A mg^−1^_Pt_). Computational studies reveal that the superior activity is associated with the presence of Ga oxides adjacent to Pt on the catalyst surface which leads to energetically favorable pathways for the oxidation process. The findings reveal untapped opportunities in the realm of liquid metal catalysis and hold great promise for the future development of high‐performance alcohol fuel cells.

## Graphical abstract

Herein, Pt–Ga liquid metal electrocatalyst has demonstrated a remarkably high mass activity towards ethanol oxidation. A low‐concentration alkaline electrolyte is formulated with guidance of machine learning neural networks. Theoretical calculation reveals that the enhanced reactivity of Pt atoms is attributed to the presence of liquid metal Ga and the surface oxides.

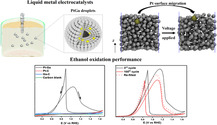

## 1 Introduction

The increasing global need for renewable energy has sparked significant interest in the development of direct ethanol fuel cells (DEFCs). In contrast to hydrogen‐based fuel cells, DEFCs do not require high pressures and are compatible with the existing fuel distribution infrastructure.^[^ ^1^, ^2^ ^]^ Ethanol is often derived from biomass and is thus a renewable and sustainable substitute for conventional fossil fuels. This factor aligns with the ongoing effort to decrease carbon emissions and transition to cleaner energy sources. Furthermore, ethanol possesses a higher theoretical energy density compared to widely utilized alternatives such as methanol and formic acid. As a result, ethanol tends to deliver a higher energy content per unit volume. In addition, ethanol is less toxic when compared to other fuel options, making it safer to handle. However, significant challenges remain that hinder the commercialization of this technology, with insufficient activity, low chemical stability, and the high cost of Pt‐containing electrocatalysts for ethanol oxidation being the main obstacles.

To date, extensive efforts have been devoted to reducing the usage of Pt in electrocatalysts while preserving their catalytic activity. This has been partly achieved by increasing the activity of Pt by adjusting the size and shape of Pt nanoparticles or by alloying Pt with lower cost metals.^[^ ^3^ ^]^ In the meantime, it has been widely recognized that the effectiveness of a catalyst is closely linked to the dispersion of metal species, which is linked to the abundance of active sites.^[^ ^4^ ^]^ As a result, diverse Pt systems spanning from nanoparticles to atomic clusters and individual atoms supported on various solid supports have been developed for DEFCs,^[^ ^5^ ^]^ most of which, however, suffer from challenging preparation processes, particle agglomeration over time, and poisoning from reaction intermediates leading to rapid deactivation of the electrocatalysts.

Recently, the low‐melting‐point metal gallium (Ga) has emerged as a catalyst and liquid support material with many intriguing features, such as low toxicity, high electric conductivity, and low melting point.^[^ ^6^ ^]^ Under ambient conditions, an ultrathin gallium oxide layer forms on its interface due to the self‐limiting oxidation of metallic Ga. This oxide layer can also actively contribute to catalytic processes.^[^ ^7^ ^]^ In addition, the ability of Ga to dissolve metal solutes across a range of concentrations can be leveraged to enhance their performance and enable their catalytic applications.^[^ ^8^ ^]^ Dissolved metal atoms can uniformly disperse in a liquid metal matrix leading to a novel form of single‐atom catalysts. Notably, a recent report showed that Pt dissolved in Ga containing only 0.0001 atomic percent of Pt exhibited a mass activity surpassing the existing commercial Pt/C catalyst for methanol oxidation.^[^ ^9^ ^]^ The dynamic Pt atoms within the Ga matrix also show minimal deactivation over time, highlighting the stability and longevity of the system. These findings have inspired this exploration of liquid metal‐based catalysts in DEFC technology.

In this work, we have developed Pt–Ga nanodroplets by dissolving 0.50 wt% Pt into liquid Ga and used them as electrocatalysts for ethanol electro‐oxidation. In contrast to bulk liquid metal alloys, the Pt–Ga nanodroplets provide higher surface area for the reaction while preserving the dynamic liquid matrix for the dispersion of Pt atoms. In order to take full advantage of this new type of catalyst, we found that the electrolyte had to be fundamentally redesigned as liquid metals can be sensitive to highly alkaline solutions used in most conventional electrolytes. In recent years, machine learning (ML) using artificial neural networks has been well integrated in material design for both electrodes and electrolytes in energy storage systems and can effectively accelerate the discovery, screening, and even prediction of materials.^[^ ^10^ ^]^ Using ML neural networks, we formulated an electrolyte with a low concentration of alkaline species (i.e., 0.03 m KOH) and optimized the concentrations of supporting electrolytes. The low alkalinity potentially mitigates the formation and precipitation of carbonates which often take place in more concentrated alkaline electrolytes.^[^ ^11^ ^]^ The reduced pH also minimizes Ga dissolution and may preserve oxide species for further surface catalytic reactions. Electrochemical benchmarking revealed that the liquid metal‐based electrodes with a low Pt loading can exhibit exceptional catalytic activity, leading to a remarkably high mass activity of Pt (13.47 A mg^−1^_Pt_) at a low peak potential of 1.03 V versus reversible hydrogen electrode (RHE). The reaction mechanisms and reaction pathways have been elucidated using computational modeling, suggesting that the presence of Ga, as well as oxide species, can effectively stabilize reaction intermediates, creating a favorable reaction pathway for ethanol oxidation.

## 2 Results and Discussion

### 2.1 Catalyst Synthesis

**Figure 1:**
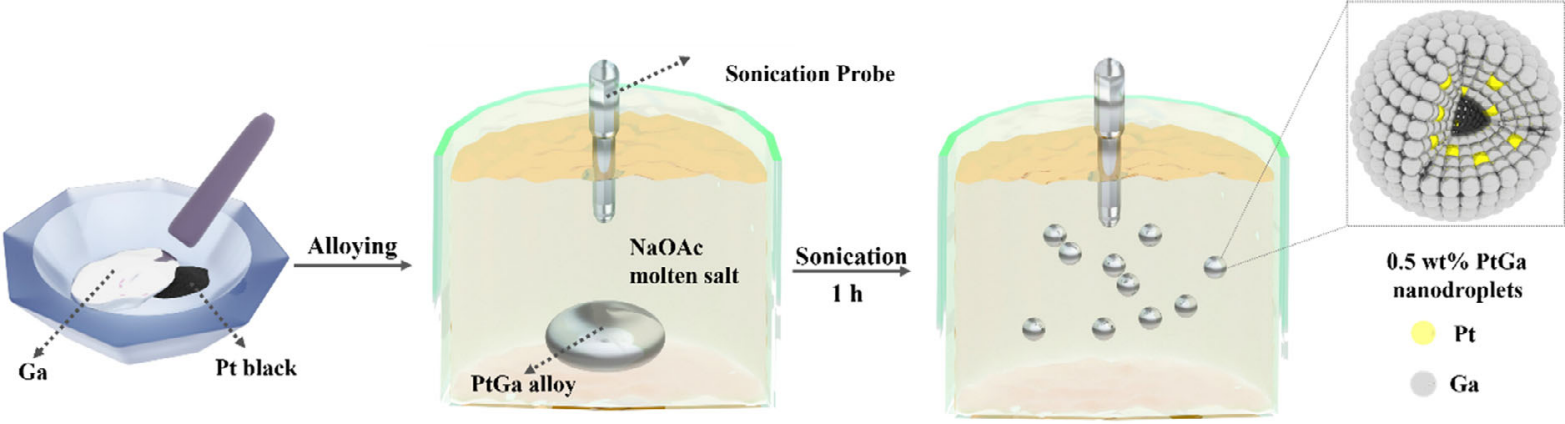
Schematic illustration of Pt–Ga alloy and nanodroplets synthesis.

The 0.50 wt% Pt–Ga nanodroplets were prepared using a previously reported high‐temperature sonication process^[^ ^12^ ^]^ (the full details are provided in the experimental section). The Pt–Ga alloy was prepared by mixing a known amount of Pt black (0.50 wt%) with Ga metal (99.5 wt%). The Pt–Ga nanodroplets were then synthesized by sonicating the Pt–Ga alloy in molten sodium acetate at 400 °C for 1 h using a probe sonicator, as illustrated in **Figure** **1**. After sonication, the nanodroplets were allowed to cool down to room temperature and washed with deionized water and ethanol to remove sodium acetate. The particles were then dried in a vacuum oven and stored for further use. The schematic illustration for the electrochemical ethanol oxidation is shown in Figure S1, Supporting Information, and the setup for the nanodroplets synthesis is shown in Figure S2, Supporting Information.

**Figure 2:**
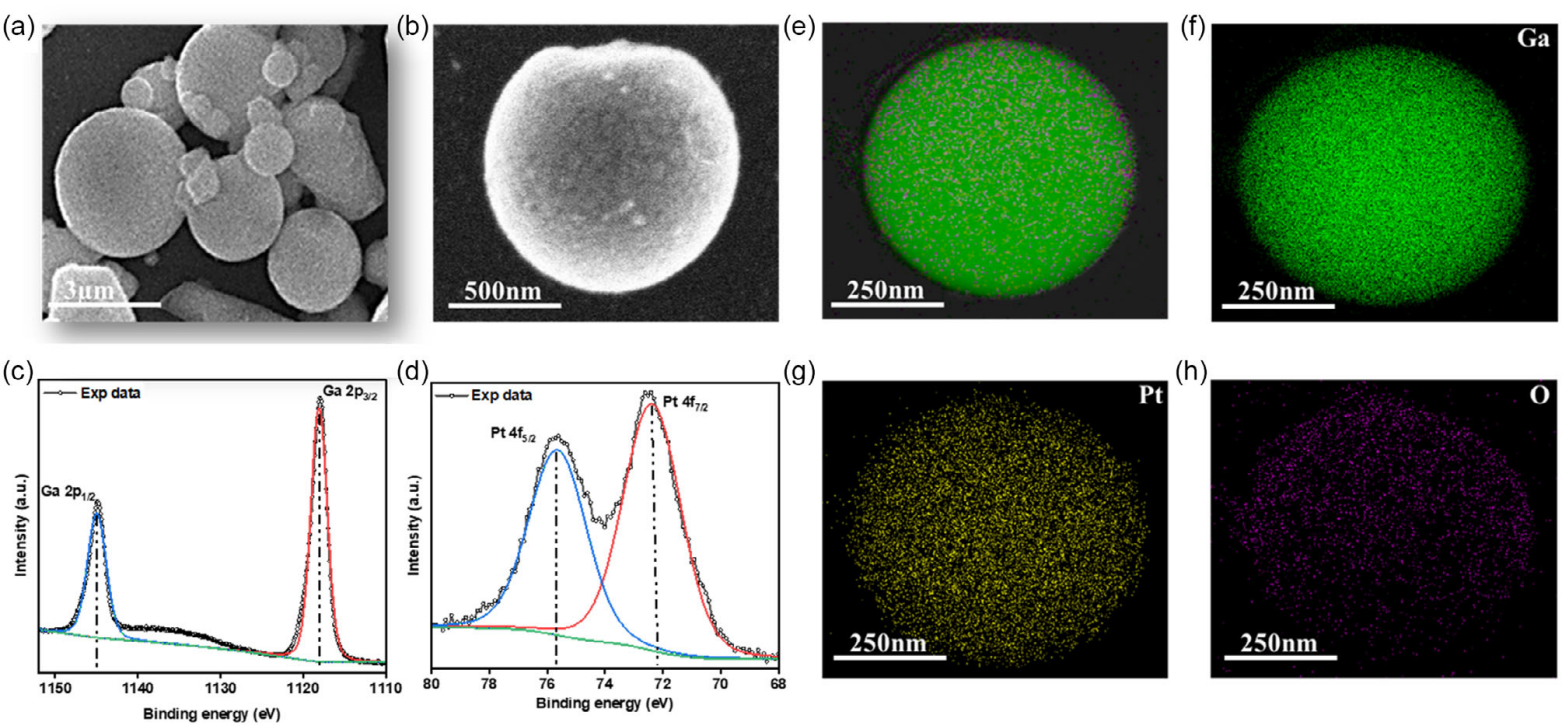
Surface and elemental analysis of the Pt–Ga system before EOR. a) SEM image of Pt–Ga nanodroplets. b) SEM image of a typical Pt–Ga nanodroplet at higher microscope magnification. c) XPS spectrum of the Ga 2*p* region. d) XPS spectrum of the Pt 4*f* region, and TEM‐based elemental maps (EDS) of a typical Pt–Ga droplet. e) Individual element maps of f) Ga, g) Pt, and h) O.

The surface morphology and elemental composition of the as‐prepared Pt–Ga nanodroplets were analyzed using scanning electron microscopy (SEM), X‐ray photoelectron spectroscopy (XPS), and energy‐dispersive X‐ray spectroscopy (EDS) mapping. As shown in **Figure** **2**a, the Pt–Ga nanodroplets are spherical with particle dimensions being on average in the range of 400 ± 193 nm but occasionally reaching a few micrometers. Figure 2b shows that the surface of the nanodroplets is very rough, likely due to the formation of gallium oxides. This works aims to take advantage of the liquid nature of gallium and its alloys at low temperatures and thus it is important to confirm that the catalyst is indeed liquid. Prior to sonication, the macroscopic bulk sample is liquid at temperatures above 30 °C and therefore the nanosized droplets are expected to remain liquid at operating conditions. This is in good agreement with published phase diagrams of the Ga–Pt system.^[^ ^13^ ^]^ To confirm the liquid state at the nanoscale, transmission electron microscopy (TEM) imaging was conducted, and the obtained images revealed the absence of crystalline solids. High‐resolution TEM imaging revealed the absence of any visible lattice structures while EDS mapping of Pt showed a homogeneous distribution (Figures S3, Supporting Information). This strongly suggests that the alloy remains liquid on the nanoscale, since previous work showed that solid intermetallics tend to be clearly visible in the TEM and are characterized by localized crystalline domains and clustering of the solute metal in EDS maps.^[^ ^12^ ^]^ To further support the assertion that most if not all Pt–Ga nanodroplets are liquid, X‐ray diffraction (XRD) was carried out at room temperature since XRD is capable of sampling many particles simultaneously rather than observing a single nanodroplet (Figure S4, Supporting Information). The XRD analysis revealed an amorphous pattern which is typical for a liquid and thus confirms that the catalyst is in a liquid state. The surface chemistry of the Pt–Ga system before ethanol oxidation reaction (EOR) was studied using XPS and the results are shown in Figure 2c,d. Ga^3+^ is the dominating species on the droplet surface, indicating the formation of gallium oxide on the surface upon exposure to air, which is consistent with the literature.^[^ ^12^ ^]^ The Ga 2*p*_3/2_ and Ga 2*p*_1/2_ peaks were observed at 1.118 and 1.144.9 eV, respectively. Observed peaks in the Pt region are discernible (Figure 2d), but noisy due to the low amount of Pt present in the sample which is approaching the detection limit of XPS. The position of the Pt 4*f*_7/2_ peak observed at 72.51 eV is in good agreement with zero‐valent Pt, while the previously reported more complex structure of Pt‐rich Ga alloys is not observed, suggesting the absence of significant concentrations of solid Pt–Ga intermetallics.^[^ ^14^ ^]^ Elemental maps of the Pt–Ga nanodroplets confirm that the individual elements are well‐dispersed without any indication of local clustering, showcasing the uniform distribution of Pt throughout the Ga matrix, as shown in Figure 2e,h.

### 2.2 Electrolyte Design

**Figure 3:**
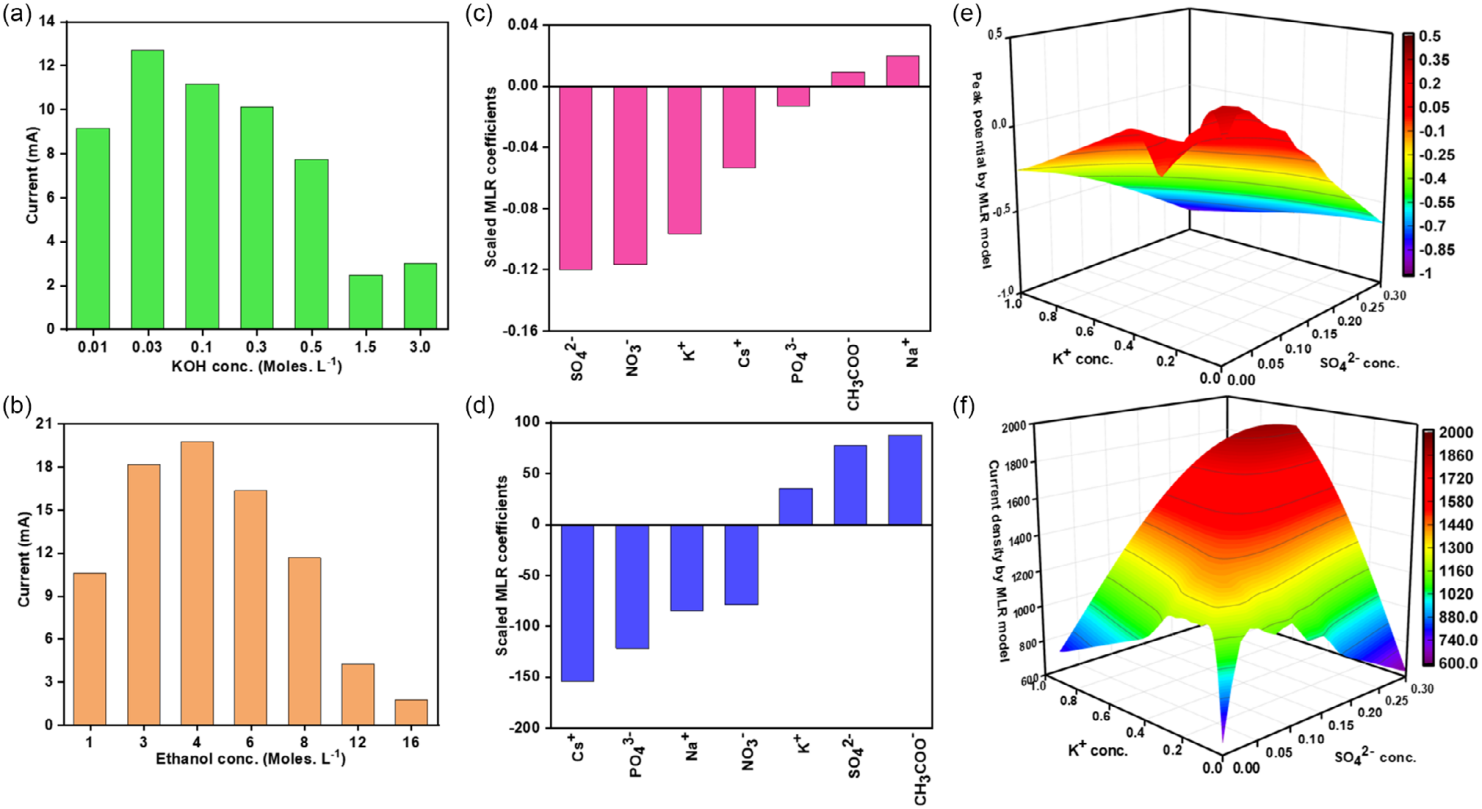
Cyclovoltammetry tests. a) Effect of KOH concentration on current. b) Effect of ethanol concentration on current. c,d) Effect of different ions on the peak potential and the current density, expressed as the scaled coefficients of the multiple linear regression model. The positive or negative sign of the coefficients indicates whether increasing the concentration of an ion will respectively increase or decrease the output value. The magnitude of the coefficients gives a measure of how large an effect the ion concentration has on the output. e,f) ML results depicting the effect of optimized SO_4_^2−^ and K^+^ concentrations on peak potential and current.

The electrolyte plays an important role in electrochemical reactions, and for DEFCs it has been reported that alkaline electrolytes are more favorable as compared to acidic ones for better catalyst stability, reduced corrosion, and minimum crossover of ethanol.^[^ ^15^ ^]^ Therefore, we selected aqueous KOH as the base electrolyte and investigated the concentration effects on the oxidation performance of the Ga–Pt electrodes. As shown in **Figure** **3**a, when the ethanol concentration is fixed at 4 m, the peak potential decreases with increased KOH concentration. This can be rationalized by considering that higher KOH concentrations provide more OH^−^ ions which can accelerate the reaction and reduce the overpotential.^[^ ^16^ ^]^ However, we observe that the peak current drops drastically when the KOH concentrations are increased above 0.50 m. This is likely because higher KOH concentrations could also cause the Ga to dissolve which compromises electrocatalysis. 0.03 m KOH was determined to be optimal through cyclic voltammetry (CV) as it resulted in the highest current with a low peak potential (CV results, Figure S5 and Table S1, Supporting Information). Based on these results, the concentration of KOH was fixed at 0.03 m in order to study the effect of ethanol concentration. The ethanol concentration was optimized using electrolytes that contained between 1 and 16 m ethanol as shown in Figure 3b (CV results, Figure S6, Supporting Information). Here, 4 m ethanol was found to be ideal. Excessive ethanol concentrations are likely to cause the blockage of active sites, hindering the adsorption of OH^−^ ions on the catalyst and thus decreasing the overall cell performance.^[^ ^17^ ^]^

The presence of supporting electrolytes and additives in the electrolyte is another crucial factor that can be considered to maximize cell performance. Alkali sulfates, nitrates, and chlorides are often used as additives to increase ionic conductivity, provide wider scan ranges, or act as cocatalysts to facilitate different reactions, for example, CO_2_ reduction as cations interact with the surface through noncovalent interactions generating strong electric fields which favor the CO_2_ reduction reaction.^[^ ^18^, ^19^, ^20^, ^21^ ^]^ Given the diverse range of concentrations and numerous possible combinations of anions and cations, electrolyte formulation can be extremely time‐consuming. Here we have developed ML models to screen and predict suitable additives to further improve the oxidation efficiency of the Pt–Ga system. The model was trained on the electrochemical data, for example, current and peak potential, collected from the electrochemical cell when electrolytes with different additives were used (Figure S7, Supporting Information). To start, we tested different types of additives with different concentrations and fed the results to the ML models. As shown in Figure 3c,d, the modeling results suggested that higher concentration of PO_4_^3−^, K^+^, Cs^+^, SO_4_^2−^, and NO_3_^−^ would provide lower peak potentials, while higher concentration of K^+^, SO_4_^2−^, and CH_3_COO^−^ tends to result in a higher current density. These modeling results were derived from a training data set containing 25 experimental entries where the concentration of ions varied from 0.025 to 0.6 m. The obtained models were used to make predictions for 144 new entries where experimental data were not available to explore the full chemical space of the ion pairs with various concentration combinations. Based on this finding, we further assessed K_2_SO_4_, KNO_3_, Cs_2_SO_4_, and CsNO_3_ and found that K_2_SO_4_ showed optimal performance in terms of oxidation current and peak potential. The model was used further to predict how the performance would change as a function of K_2_SO_4_ concentration, as shown in Figure 3e,f. While conducting experimental verifications, we found that the solubility of K_2_SO_4_ was limited in the optimized ethanol/KOH solution. In the end, we selected 100 mm K_2_SO_4_ as the optimum concentration. It is worth noting that the predictive model does not apply to electrolytes containing multiple metal salts, due to the unavailability of such experimental data for training. This will be explored in future work where additional experimental results associated with the solubility and ionic conductivity will be generated for training ML models and enable accurate prediction for multicomponent systems.

**Figure 4:**
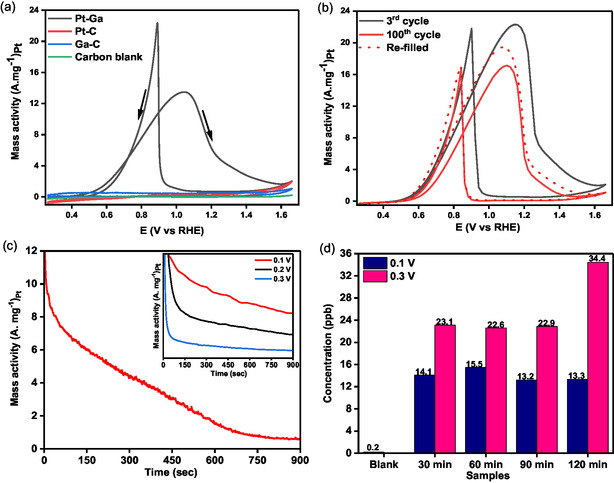
Electro‐oxidation performance of ethanol in an alkaline media. a) EOR‐specific activity of 0.5 wt% Pt–Ga, Ga–C, and the commercial Pt/C in 0.03 m KOH with 4 m ethanol solution. b) Comparison of EOR performance of Pt–Ga between 3^rd^ and 200^th^ cycles. c) Comparison of CA analysis of Pt–Ga system at different voltages (vs Ag/AgCl). d) ICP‐MS analysis of electrolytes cycled at 0.1 and 0.3 V vs Ag/AgCl for 2 h.

With the formulated electrolyte, that is, 0.03 m KOH and 4 m ethanol with 100 mm K_2_SO_4_, we carried out systematic electrochemical testing of the Pt–Ga electrocatalysts. A typical electrode was made from a piece of 1 cm  × 1 cm carbon paper with the droplets deposited onto its surface. For comparison, a Pt/C catalyst with the same loading of Pt onto carbon powder was prepared and tested for EOR. The CV results of Pt‐Ga/C, Pt/C, and Ga/C are shown in **Figure** **4**a. The onset potential of Pt‐Ga/C is 0.42 V versus RHE, which is much lower than values reported in previous studies, confirming that Pt–Ga is an excellent catalyst for ethanol oxidation.^[^ ^22^, ^23^ ^]^ The Pt/C and Ga/C catalyst, however, showed negligible activity in the electrolyte we used for PtGa nanodroplets. The low activity of Pt/C might originate from two factors. First, the Pt‐Ga/C and Pt/C were prepared with a Pt loading which is significantly lower than those used in commercial Pt/C catalysts. Another reason is the electrolyte used which was optimized for the liquid alloy catalysts developed in this study and its chemical composition is significantly different from those of conventional high‐concentration KOH‐based electrolytes. When tested in a conventional electrolyte, that is, 1 m ethanol and 0.5 m KOH, the maximum mass activity of Pt/C was found to be 2.17 A mg^−1^_Pt_ at a voltage of 1.005 V versus RHE which is almost 7 times lower than the activity of liquid Pt–Ga catalyst (Figure S8, Supporting Information). This not only demonstrates the superior activity of the Pt–Ga catalyst but also highlights the importance of reformulating the electrolyte to achieve optimum performance from different catalyst systems.

The peak potential in the forward scan for the Ga–Pt nanodroplets was observed at 1.03 V versus RHE and a peak between 0.95 and 0.56 V versus RHE emerged during the reverse scan and can be ascribed to the oxidation of intermediates formed during ethanol oxidation in the forward scan. The electrode area's normalized activity of the electrode toward ethanol oxidation was then determined to be 3.4 mA cm^−2^ (see Figure S9 and S10, Supporting Information, for electrochemical active surface area [ECSA] calculation), and the mass activity was as high as 13.47 A mg^−1^_Pt_. This value is almost 14 times higher than that obtained from commercial Pt/C catalysts and higher than those reported in the previous studies (Table S2, Supporting Information).^[^ ^24^, ^25^ ^]^ To study the durability of the Pt–Ga system, CV scans were obtained at a scan rate of 100 mV s^−1^ for the 3^rd^ and the 100^th^ subsequent cycles, as shown in Figure 4b. Overall, the performance remained high after 100 cycles. Following the 100^th^ cycle, we refilled the cell with fresh electrolyte and ran another 10 cycles. The results showcase that after refilling the electrolyte, 87% of the initial activity can be recovered, indicating excellent stability of the Pt–Ga electrode. Furthermore, chronoamperometry (CA) tests at different voltages and times were performed to investigate the stability of the Pt–Ga system. As shown in Figure 4c, at a lower voltage of 1.06 V versus RHE, the Pt–Ga electrode showed good stability for 900 s. On the contrary, at higher voltages of 1.16 and 1.26 V versus RHE, the performance decreased rapidly. This might be due to the higher oxidation voltage which accelerated the dissolution of Ga and caused a reduction of reactive sites on the electrodes.^[^ ^26^ ^]^ This hypothesis is supported by inductively coupled plasma‐mass spectrometer (ICP‐MS) results, where higher concentrations of Ga species were found in the electrolyte when a more oxidizing voltage was applied (Figure S11, Supporting Information). As shown in Figure 4d, after cycling at 1.06 V versus RHE for 2 h, the concentration of Ga in the electrolyte is 13.3 ppb, remaining almost constant from 30 to 120 min, while for the electrolyte cycled at 1.26 V versus RHE, the concentration of Ga was found to be 34.4 ppb after 2 h which is almost 2.5 times higher. The Ga concentration remained unchanged when a lower voltage was applied, indicative of the stability of the electrodes.

**Figure 5:**
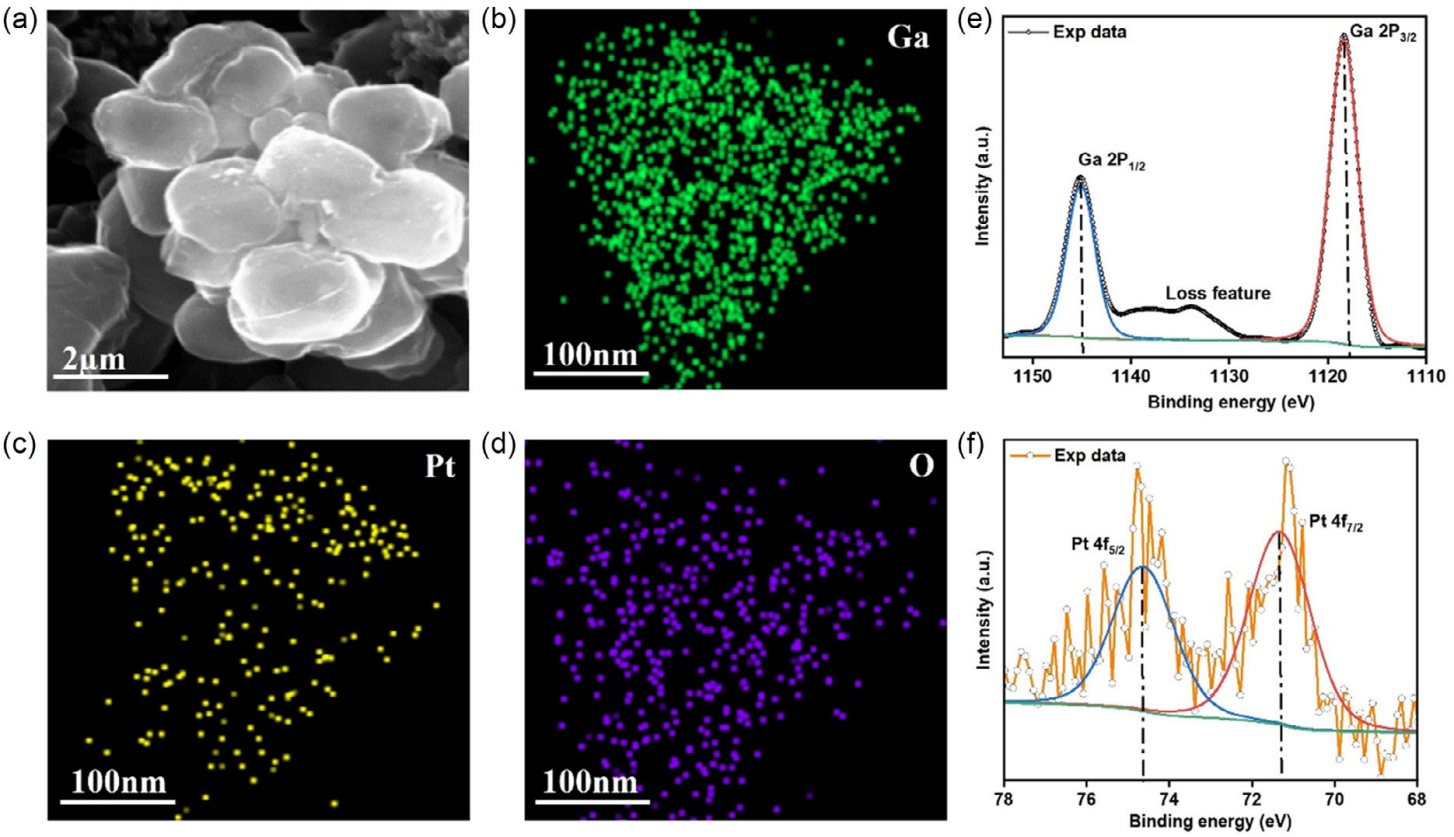
Surface and elemental analysis of Pt–Ga system after EOR. a) SEM image of Pt–Ga nanodroplets on carbon electrode. b–d) Elemental mapping images of Ga, Pt, and O. e) XPS spectrum of Ga 2*p* of Pt–Ga system and f) XPS spectrum of Pt 4*f* of Pt–Ga system.

Surface chemistry, morphology, and elemental composition analysis of Pt–Ga after EOR are presented in **Figure** **5**. As shown in Figure 5a, there was a minor change in the morphology of the Pt–Ga nanodroplets transforming from well‐defined spherical particles into more irregular particles, which might be due to the oxidation of the particles during the reaction process. Elemental maps (Figure 5b–d) of the Pt–Ga nanodroplets confirmed the presence and dispersion of Pt on the Ga surface after EOR, showing the stability of the Pt–Ga system. The surface chemistry was studied using XPS and is shown in Figure 5e,f. The Ga 2*p*_3/2_ and Ga 2*p*_1/2_ peaks observed in the XPS spectrum were positioned at 1,116.98 and 1,144.68 eV and can be attributed to Ga oxide (Figure 5e). The Pt 4*f*_7/2_ and Pt 4*f*_5/2_ peaks at 71.08 and 74.78 eV can be ascribed to zero‐valent Pt (Figure 5f). The negative shift of binding energies for Pt after EOR might be due to the electron charge transfer from Ga to Pt. As reported in previous studies,^[^ ^14^, ^27^ ^]^ with the increase of the adjacent Ga atoms, the electron cloud density of Pt increases so that the spectra corresponding to Pt (0) move to the direction of lower binding energy. This indicates that Ga or its oxide actively contributes to the reaction process and stabilizes the metallic state of Pt during EOR. The reduced peak intensity when compared to the results shown for as‐prepared catalysts is most likely due to the sample preparation and the fact that used particles are embedded as a sparse coating on carbon paper when measured rather than a dense drop cast powder. As such, the intensity should not be interpreted in this case. The peak positions overall remained unchanged however, showcasing that the oxidation states of both Ga and Pt at the interface did not appear to be changed. It is worth noting that despite the fact that Ga_2_O_3_ is dominantly presented on its surface, the PtGa catalyst is distinctive from the Ga_2_O_3_‐supported Pt (Pt/Ga_2_O_3_) catalyst system. In our case, the Pt is dissolved in the liquid metal. Hence the liquid Ga is more than just a support and instead results in unique properties. Since the Pt is dissolved, atomically segregated active sites in a liquid metal environment are achieved which improve activity and selectivity. For comparison, a sample of Pt supported on commercially sourced Ga_2_O_3_ was prepared and tested for EOR. The EOR was performed in an electrolyte having 4 m ethanol, 0.03 m KOH, and 100 mm K_2_SO_4_ at 70 °C at a scan rate of 20 mV s^−1^. The CV results of Ga_2_O_3_‐supported Pt for EOR are shown in Figure S12, Supporting Information. The maximum mass activity of 1.39 A mg^−1^_Pt_ was obtained at 0.93 V versus RHE which is much lower when compared with liquid Pt–Ga catalyst for ethanol oxidation under the same conditions. This further highlights the superiority of using liquid metal rather than Ga_2_O_3_ as support for the Pt catalyst.

To elucidate the reaction mechanism, we further analyzed the products from the reaction. During the oxidation reaction, the production of gas bubbles was observed. The composition of these gas bubbles was analyzed using head space gas chromatography, revealing that H_2_ was the main component of the gas. We also performed ^1^H analyses to identify any liquid products that may have been produced from the ethanol oxidation reaction (Figure S13, Supporting Information). The ^1^H NMR analysis confirmed the formation of acetaldehyde and acetate after EOR. The peaks at 1.03, 3.48, and 4.64 ppm can be attributed to the presence of ethanol and water in the electrolyte.^[^ ^28^, ^29^ ^]^ The peak at 1.75 ppm confirms the presence of acetate whereas the peaks at 2.3 and 8.3 ppm indicate the formation of acetaldehyde and dialdehyde, respectively.^[^ ^30^, ^31^ ^]^

**Figure 6:**
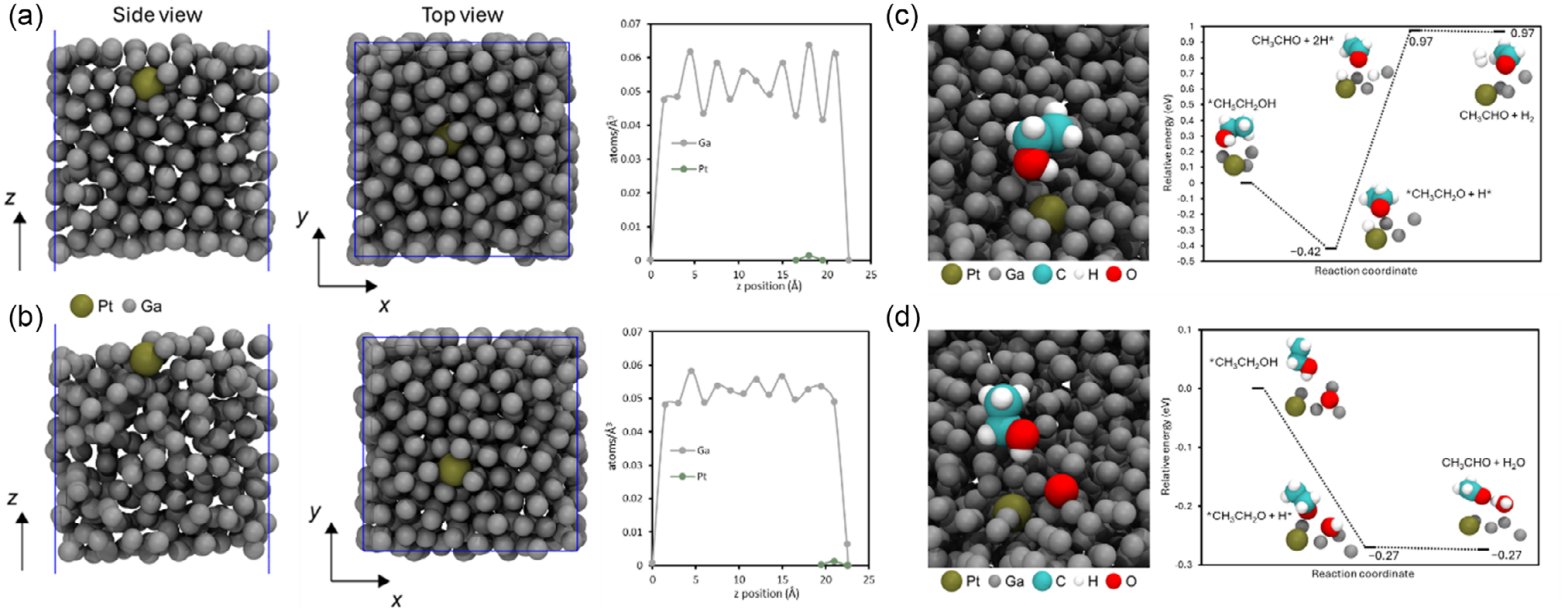
Side view (cross‐section), top view, and atomic density profiles for systems a) run with no perturbation (top) and b) with an electric field of −0.13 V Å^−1^ (bottom). All simulations were run for 10 ps. c) Reduction of ethanol to acetaldehyde by direct transfer of H from the ethanol hydroxyl group to Pt. All energies are shown relative to the initial reactants. d) Reduction of ethanol to acetaldehyde by transfer of H from the ethanol hydroxyl group to a surface oxygen. All energies are shown relative to the initial reactants.

Ab initio molecular dynamics (AIMD) simulations were performed to investigate the catalytic reaction and elucidate the reaction mechanisms. In our previous work, we found that for Pt in Ga, the Pt was found one atomic layer below the interface in the spontaneous oxidation of pyrogallol^[^ ^32^ ^]^ but directly at the interface for the electrooxidation of methanol.^[^ ^32^, ^33^ ^]^ In order to reconcile this apparent discrepancy, we ran AIMD simulations of 499 Ga and one Pt (i.e., 0.5 wt%) without any perturbation and with an electric field of −0.13 V Å^−1^, corresponding to an absolute potential of 4.6 V, in the z dimension, to represent the potential of the cell in use. We also ran simulations with one electron removed from the system to simulate an electron‐deficient anode. The results are presented in **Figure** **6**a,b.

Similar to our previous findings, the Pt atom was found at least one atomic layer below the surface in the absence of any perturbation to the system. In simulations of up to 100 ns, the Pt atom was consistently found 1–2 layers below the interface, but never in the outermost interfacial layer. In contrast, in electron‐deficient systems and systems with an electric field, the Pt atom is found in the interfacial layer and sufficiently exposed at the interface to react directly with ethanol. This is in line with the findings of Baharfar et al.^[^ ^34^ ^]^ who showed that applying an electric field can expel more electronegative elements from liquid Ga.

For the reduction of ethanol to acetaldehyde, two H atoms must be removed from ethanol: from the hydroxyl O atom and the adjacent methylene C atom. To explore this reaction in more detail at the Ga–Pt interface, we first modeled a process analogous to the one described previously for methanol: the hydroxyl H is transferred to Pt, followed by the transfer of a methylene H to an adjacent Ga (Figure 6c). For this reaction, the intermediate following the transfer of the hydroxyl H from ethanol to the surface Pt is lower in energy by −0.42 eV, relative to the reactants, while the second step, where a second H is transferred from the methylene C to Ga, is ≈1 eV higher in energy. Additionally, from the position of the first intermediate, where the ethanol O is stabilized by a surface Ga adjacent to Pt, it is unclear what the pathway would be for the methylene H to transfer to a surface Ga. Moreover, the energy relative to the reactants for forming H_2_ rather than two H on the surface is virtually identical. Based on previous studies, we would expect the reaction to become more energetically favorable under an applied electric field^[^ ^35^ ^]^ but for this reaction, the first step actually increased in energy slightly, to −0.35 eV, while the second step only decreased slightly, to 0.96 eV. An alternative for the final step would be the transfer of H from the methylene C to a hydroxide ion in solution, resulting in water and acetaldehyde as products.

Another potential mechanism involves the presence of O (i.e., gallium oxide) on the Ga–Pt surface. In this mechanism, the hydroxyl H of ethanol is transferred to a surface O atom adjacent to Pt, and similar to the previous mechanism, the ethanol O is stabilized by Ga adjacent to Pt (Figure 6d). In this mechanism, the first step is energetically favorable by −0.27 eV, while the second step, which releases both acetaldehyde and water from the surface, is energetically equivalent (relative to the initial reactants). In contrast to the previous mechanism, when an electric field is applied, both steps become more energetically favorable, with the first step increasing to −0.28 eV and the second to −0.34 eV. Similar to the previous mechanism, the second step could also involve the transfer of H from the methylene C to a hydroxide ion in solution, leaving OH on the surface that could react further with acetaldehyde to produce acetic acid. Both mechanisms indicate that the presence of Ga and the oxides on the surface play a critical role in determining the reaction products and pathways with the Ga‐based electrodes. In the future we will investigate the reaction mechanism further experimentally utilizing techniques such as operando X‐ray absorption spectroscopy.

## 3 Conclusion

A highly efficient and stable liquid metal‐based electrocatalyst has been developed to promote ethanol oxidation. A low‐concentration alkaline electrolyte has been specially formulated with the aid of ML model. The liquid metal‐based electrodes have demonstrated exceptional activity and stability toward ethanol oxidation with a mass activity of Pt species more than 10 times higher than that of a commercial catalyst. Density functional theory modeling indicates that the favorable reaction pathways were largely attributed to the presence of oxides adjacent to Pt at the surface. In future, we are aiming to develop more efficient liquid metal catalysts to promote the full oxidation of ethanol by tailoring the composition of the liquid metal alloys or introducing more metal species into the liquid metal matrix to create more energetic and active sites to facilitate the bond formation/dissociation of reactants in the EOR process. The findings may open new avenues to guide the design and fabrication of high‐performance DEFCs.

## 4 Experimental Section

4.1

4.1.1

##### Materials

All the materials used including Pt, Ga, ethanol, sodium acetate, potassium sulfate, potassium nitrate, sodium sulfate, sodium nitrate, cesium acetate, sodium acetate, and cesium sulfate of analytical grades (99.9% purity) were purchased from Sigma Aldrich Australia.

##### Pt–Ga Alloy & Nanodroplets Synthesis

Pt (0.50 wt%)–Ga alloy was prepared by mixing 0.10 g of platinum black nanopowder in 19.90 g of gallium metal at 350 °C inside a glovebox to avoid oxidation. This temperature was selected based on the binary phase diagram of Pt–Ga system.^[^ ^36^ ^]^ The metals were mixed using a mortar and pestle until the complete homogenized alloy was formed after about 60 min. Later, the alloy was cooled down to room temperature and stored in a freezer at −80 °C. After solidification, the alloy was cut down into small chunks and stored in an airtight container at 4 °C.

Pt–Ga nanodroplets were synthesized by taking 1.8 g of Pt–Ga alloy and breaking it down using a probe sonicator at 400 °C in molten sodium acetate (15.8 g). Probe sonication was conducted for 30 min using a SCIENTZ‐IID probe sonicator, equipped with a 6 mm titanium tip at 300 W power. The reaction temperature was maintained on a hotplate inside a specially machined aluminum heating block and monitored using a thermocouple (Figure S2, Supporting Information).^[^ ^12^ ^]^ A schematic illustration of the nanodroplet synthesis process is shown in Figure 1. After sonication, nanodroplets were allowed to cool down to room temperature and were then washed and filtered using deionized water and ethanol solution. The final product was dried in a vacuum oven at 40 °C for 2 h to remove remaining moisture and ethanol and then stored for later use.

##### Catalyst Characterization

SEM was performed through FEI Verios 460 L XHR‐SEM at an operating pressure of 5 × 10^−6^ mbar, 3 kV accelerating voltage, 100 pA current, and ≈12 k magnification. Magnification values varied slightly depending on the focus of the image with the scale remaining standard. SEM–EDS images and data were obtained using the SEM device equipped with an Oxford Instruments Xmax EDS detector. To avoid unnecessary element detection, each system was drop cast onto a pure Si wafer and data was processed using Aztec software. TEM analysis was performed using TEM, JEOL F200, at an accelerating voltage of 200 kV. Gatan Digital Micrograph 3.43.3213.0 software suite was used for imaging and analysis with the use of Gatan Rio16 4 k charge‐coupled device camera (model 1816). EDS was used on Aztec software with the attached EDS Oxford X‐Maxn 80 T X‐ray spectrometer. XPS analysis was performed using a Thermo Scientific K‐alpha XPS spectrometer X‐ray source (monochromatic Al K‐alpha source, *hv* = ≈1,486.6 eV). The instrument was equipped with a concentric hemispherical analyzer. The XPS data was analyzed through Casa‐XPS software (version 2.3.25PR1.0). XRD patterns were obtained using a Bruker D4 diffractometer with Cu Kα radiation (1.5418 Å).

Proton nuclear magnetic resonance (^1^H NMR) technique was performed using Bruker Avance III 300 MHz NMR spectrometer equipped with a 5 mm BBO probe. Samples were prepared by mixing 540 μL of electrolytes with 60 μL of D_2_O for NMR analysis. ICP‐MS analysis was performed in ICP‐MS Agilent 7700 with MicroMist nebuliser and X‐lens by different electrolytes after EOR in aqua regia.

##### Electrochemical Test of Ethanol Oxidation

Catalytic activity of Pt–Ga nanodroplets for electrochemical ethanol (C_2_H_5_OH) oxidation was tested in KOH solution, using Pt‐Ga/C as working electrode, platinum wire as counter electrode, and Ag/AgCl as reference electrode. CV analysis was performed using Vertex.C.EIS potentiostat (350 mA/13 V/1 MHz). To make the working electrode, 100 μL Pt–Ga nanodroplets (≈0.00175 mg of Pt) and Ga droplets dispersed in ethanol were drop cast onto carbon papers and dried before using for ethanol oxidation. The Pt loading was equivalent to 0.015 wt% in a typical carbon electrode. CV tests were carried out at 70 °C from −0.70 to 0.70 V versus Ag/AgCl at a scan rate of 20 mV s^−1^ with varying concentrations of ethanol, KOH, and additives. For the ECSA measurement, the CV was performed in N_2_‐saturated 0.5 m H_2_SO_4_ solution over a potential range from −0.15 to 1.05 V (vs. Ag/AgCl) at a scan rate of 50 mV s^−1^ and referred to as RHE.^[^ ^1^ ^]^ The value of ECSA of catalysts was estimated from the average of hydrogen adsorption/desorption adsorption region between −0.15 and 0.20 V (vs. Ag/AgCl) after correcting for double‐layer charging current in the voltammogram. The value of ECSA was calculated based on the following equation.

(1) ECSA=QH/m × qH

where QH (μC) is the average charge of hydrogen adsorption/desorption, *m* (μg) is the Pt metal loading, and *qH* (μC cm^−2^) is the charge for desorbing a monolayer of hydrogen on a Pt surface, where *qH* of 210 μC cm^−2^ was used. The ECSA measurement curve is shown in Figure S9, Supporting Information, and the electrode area normalized activity is given in Figure S10, Supporting Information.

The results of CV were then presented versus RHE utilizing the following equation.

(2) $$E_{\text{RHE}}=\;E_{\text{Ag/AgCl}}+\;0.224\;+\;0.059\;\left(\text{pH}\right)$$

##### ML for Electrolyte Formulation

Multiple linear regression and Bayesian‐regularized artificial neural networks implemented in the in‐house BioModeller package were used in this study.^[^ ^37^, ^38^, ^39^ ^]^ The inputs of the ML models consisted of seven variables corresponding to the concentrations of the different ions (sodium, potassium, cesium, sulfate, nitrate, phosphate, and acetate). The output of the models was the peak potential or the current. The artificial neural network had three layers, consisting of input, hidden, and output layers. There were seven neurons in the input layer, corresponding to the concentration of the 7 ions. The hidden layer had two neurons while the output layer had one neuron corresponding to the peak potential or the current density. 25 data entries where the concentration of ions varied from 0.025 to 0.6 were used to train the models and the obtained models were used to make predictions for 144 new entries to explore the full chemical space of the ion pairs with various concentration combinations.

Predictions were also made for 1,248 new cases where there were multiple cations and anions in the systems. However, as the training data only contained samples with single cation and single anion, the predictions for multiple cations and anions must be used with care.

Training data as well as predictions generated by the obtained models are provided in the Supporting Information.

##### MD Simulations

Initial classical molecular dynamics (MD) simulations were performed with 500 Ga atoms in a 21.228 × 21.228 × 21.228 Å^3^ box using the MD code LAMMPS^[^ ^40^ ^]^ in order to rapidly generate equilibrated configurations of the liquid metal. Force field parameters for Ga were taken from our previous work.^[^ ^41^ ^]^ Following this initial equilibration, one surface Ga atom was alchemically converted to Pt atom (0.5 wt% Pt). A 15 Å vacuum spacer was added in the *z* dimension and interfacial AIMD simulations were performed on this system for 10 ps with a 4 fs timestep using the Vienna ab initio Simulation Package^[^ ^42^, ^43^ ^]^ at 343.15 K with the projector‐augmented wave^[^ ^44^ ^]^ method, the Perdew–Burke–Ernzerhof exchange correlation functional,^[^ ^45^ ^]^ an energy cutoff of 250 eV, and the gamma point only for the *k*‐point grid. For simulations involving ethanol, random initial configurations of ethanol were added to equilibrated snapshots of the liquid metal interface. The timestep was reduced to 1 fs and the energy cutoff increased to 420 eV. To generate the reaction profile, multiple configurations were examined dynamically in a procedure similar to that outlined by Ruffman et al.^[^ ^33^ ^]^ Geometry optimizations were performed on AIMD snapshots for each step in the reaction with a 4 × 4 × 1 *k*‐point grid. All other analysis was performed using VMD 1.9.3.^[^ ^46^, ^47^ ^]^

## Conflict of Interest

The authors declare no conflict of interest.

## Author Contributions

**Muhammad Hamza Nazir**: Writing—original draft (lead). **Tu C. Le**: Investigation (supporting); Methodology (supporting). **Imtisal Zahid**: Writing—review & editing (supporting). **Karma Zuraiqi**: Validation (supporting). **Mew P. Aukarasereenont**: Data curation (supporting). **Caiden J. Parker**: Resources (supporting); Validation (supporting). **Pierre H. A. Vaillant**: Data curation (supporting). **Fahad Jabbar**: Visualization (equal). **Chung Kim Nguyen**: Data curation (supporting); Resources (supporting). **Mehmood Irfan**: Data curation (supporting). **Mariam Ameen**: Resources (supporting). **Michelle J. S. Spencer**: Conceptualization (supporting). **Andrew J. Christofferson**: Conceptualization (supporting); Data curation (supporting). **Salvy P. Russo**: Data curation (supporting). **Ken Chiang**: Conceptualization (equal); Project administration (equal). **Nastaran Meftahi**: Data curation (supporting); Methodology (supporting). **Torben Daeneke**: Conceptualization (equal); Supervision (equal). **Dan Yang**: Supervision (equal); Writing—review & editing (supporting).

## Supporting information

Supplementary Material

## Data Availability

The data that support the findings of this study are available from the corresponding author upon reasonable request.
