# Supplementary material for: Liquid Metal Electrocatalyst with Ultralow Pt Loading for Ethanol Oxidation
Source: Small Sci. 2024 Oct 6;5(1):2400370. doi: 10.1002/smsc.202400370 (PMC11935015; doi:10.1002/smsc.202400370)
Supplement: Supplementary file 1 — Supplementary Material [file SMSC-5-2400370-s001.pdf]

Liquid Metal Electrocatalyst with Ultra-low Pt Loading for Ethanol Oxidation

Supporting Information

Muhammad Hamza Nazir[a], Tu C. Le[b], Imtisal Zahid[a], Karma Zuraiqi[a], Mew P Aukarasereenont[a], Caiden J. Parker[a], Pierre H.A. Vaillant[c], Fahad Jabbar[a], Chung Kim Nguyen[a], Mehmood Irfan[a], Mariam Ameen[a], Michelle J. S. Spencer[c], Andrew J. Christofferson[c], Salvy P. Russo[c], Ken Chiang[a*], Nastaran Meftahi[c*], Torben Daeneke[a*] and Dan Yang[a*]

[a] Department of Chemical and Environmental Engineering, School of Engineering, RMIT University, Melbourne, VIC 3001, Australia

[b] Department of Manufacturing, Materials and Mechatronics, School of Engineering, RMIT University, Melbourne, VIC 3001 Australia

[c] School of Science, RMIT University, Melbourne, VIC3001 Australia

Corresponding Authors: ken.chiang@rmit.edu.au, nastaran.meftahi@rmit.edu.au, torben.daeneke@rmit.edu.au, dan.yang@rmit.edu.au

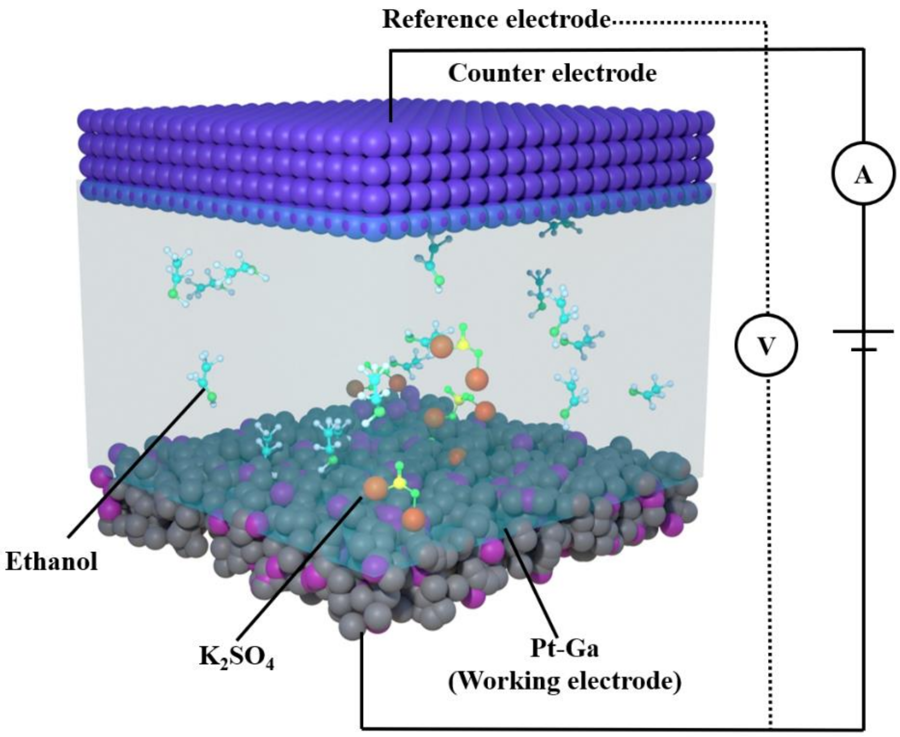

Figure S1. Schematic illustration of the electrochemical setup for ethanol oxidation.

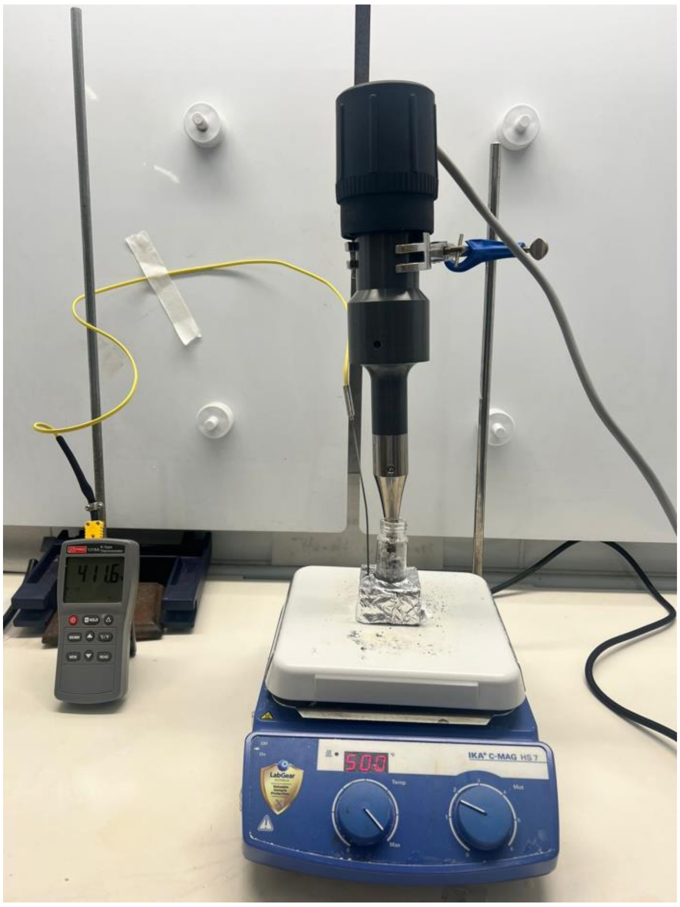

Figure S2. Setup for nanodroplets synthesis.

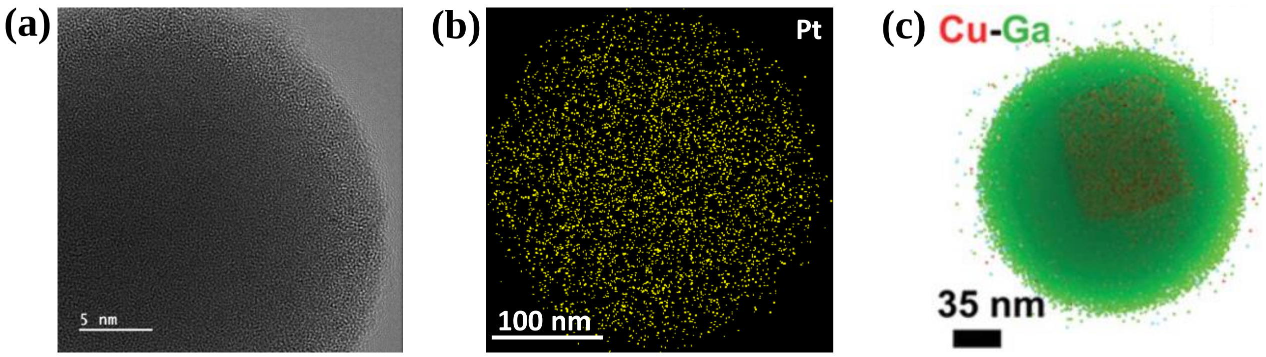

Figure S3. (a) High-resolution TEM of 0.5 wt% Pt-Ga, and (b) EDS mapping of Pt in Pt-Ga, and (c) An overlayed EDX elemental map of 5 wt% Cu-Ga, clearly showing the presence of a solid intermetallic particle. This image is provided as a reference point as to how a liquid metal nanodroplet looks that has partially solidified. Reproduced with permission from [1], Copyright 2023, WILEY Online Library.

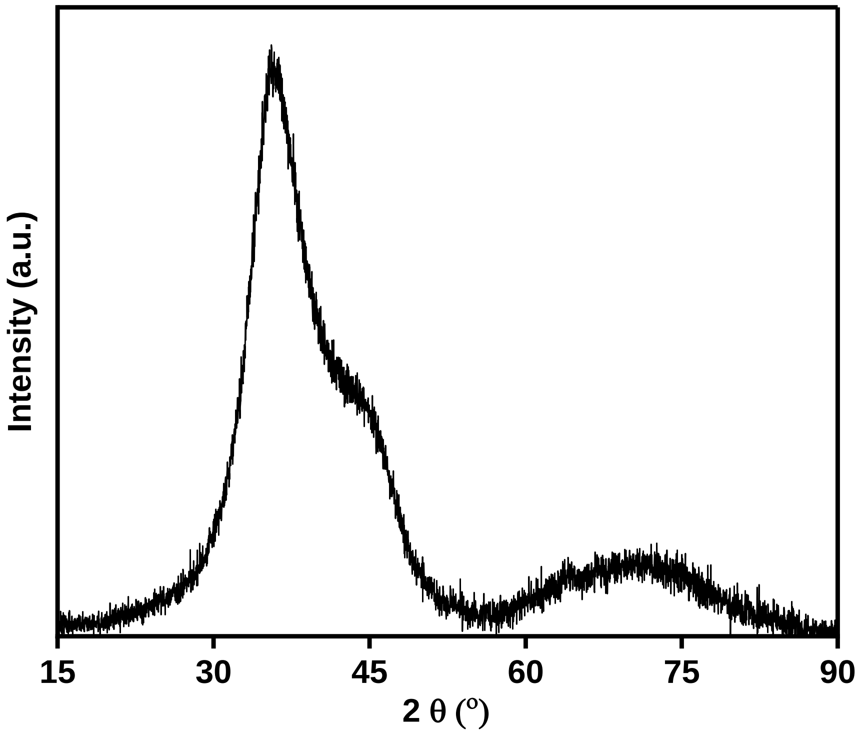

Figure S4. XRD pattern of 0.5 wt% Pt-Ga catalyst.

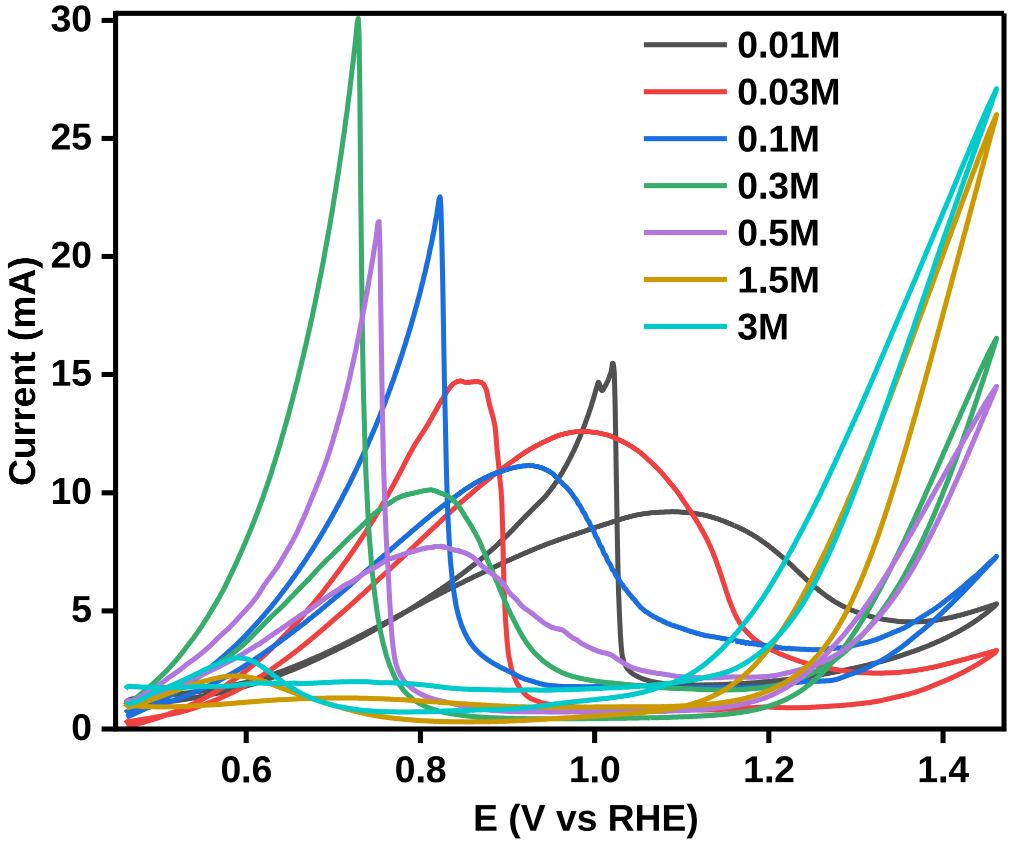

Figure S5. CV results of different KOH concentrations on current and peak potential.

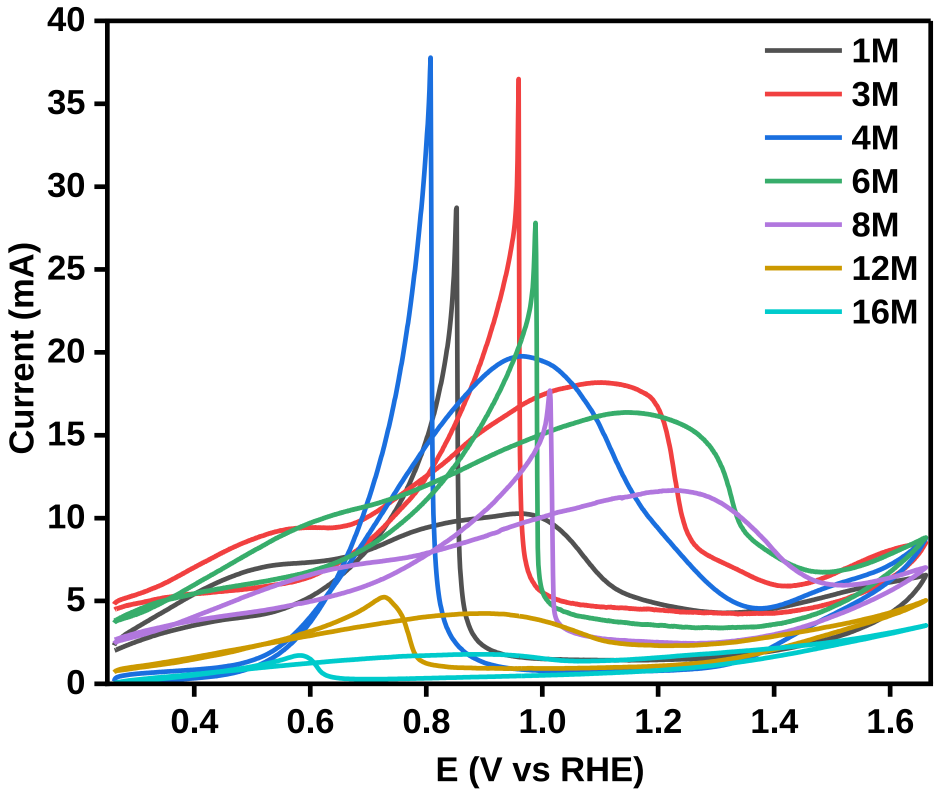

Figure S6. CV results of effect of ethanol concentrations on peak potential and current.

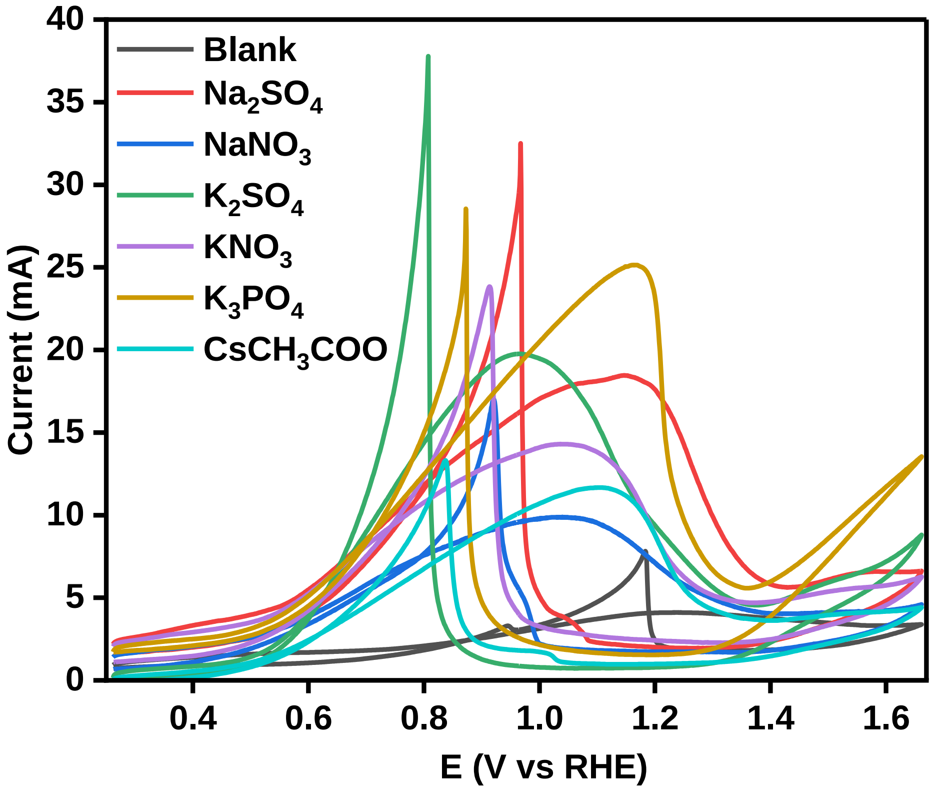

Figure S7. CV results of effect of different additives on peak potential and current.

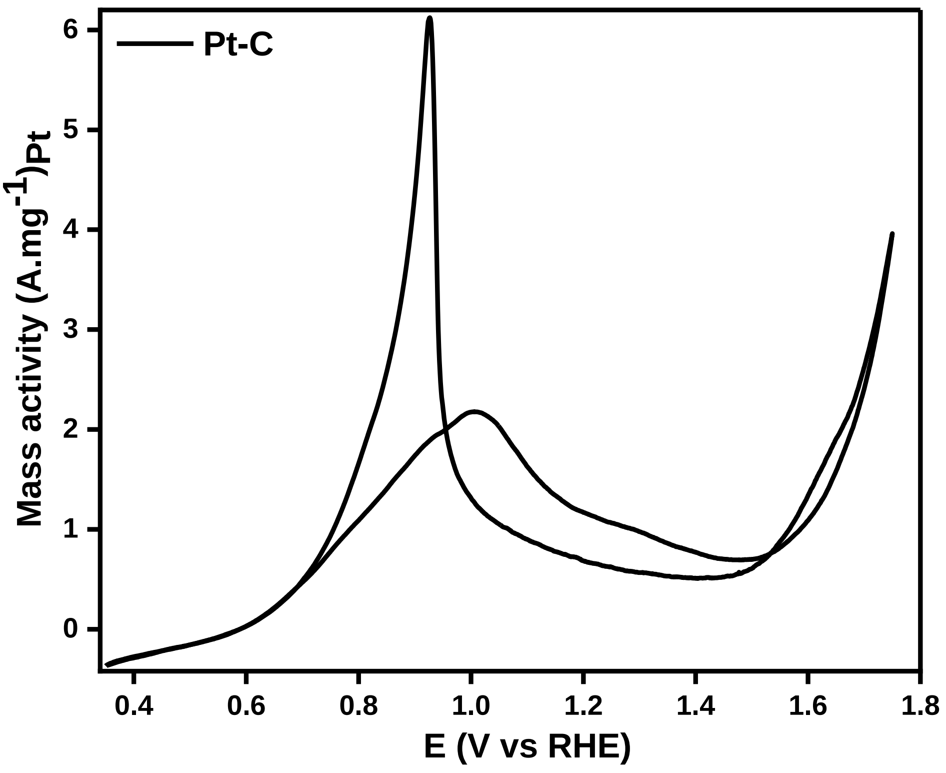

Figure S8. Electrochemical oxidation of ethanol using Pt-C catalyst.

For the electrochemical active surface areas (ECSA) measurement, the cyclic voltammetry was performed in N_2-saturated 0.5 M H_2SO_4 solution over a potential range from -0.15 V and 1.05 V (vs. Ag/AgCl) at a scan rate of 50 mV/s and referred to as RHE [2]. The value of ECSA of catalysts was estimated from the average of hydrogen adsorption/desorption adsorption region between -0.15 V and 0.20 V (vs. Ag/AgCl) after correcting for double layer charging current in the voltammogram as shown in Figure S7. The value of ECSA was calculated based on the following equation:

$$ECSA = QH/m \times qH$$

Where QH (μC) is the average charge of hydrogen adsorption/desorption, m (μg) is the Pt metal loading, and qH (μC/cm^2) is the charge for desorbing a monolayer of hydrogen on a Pt surface, with qH of 210 μC/cm^2 is used. The electrode activity based on area is given in Figure S9.

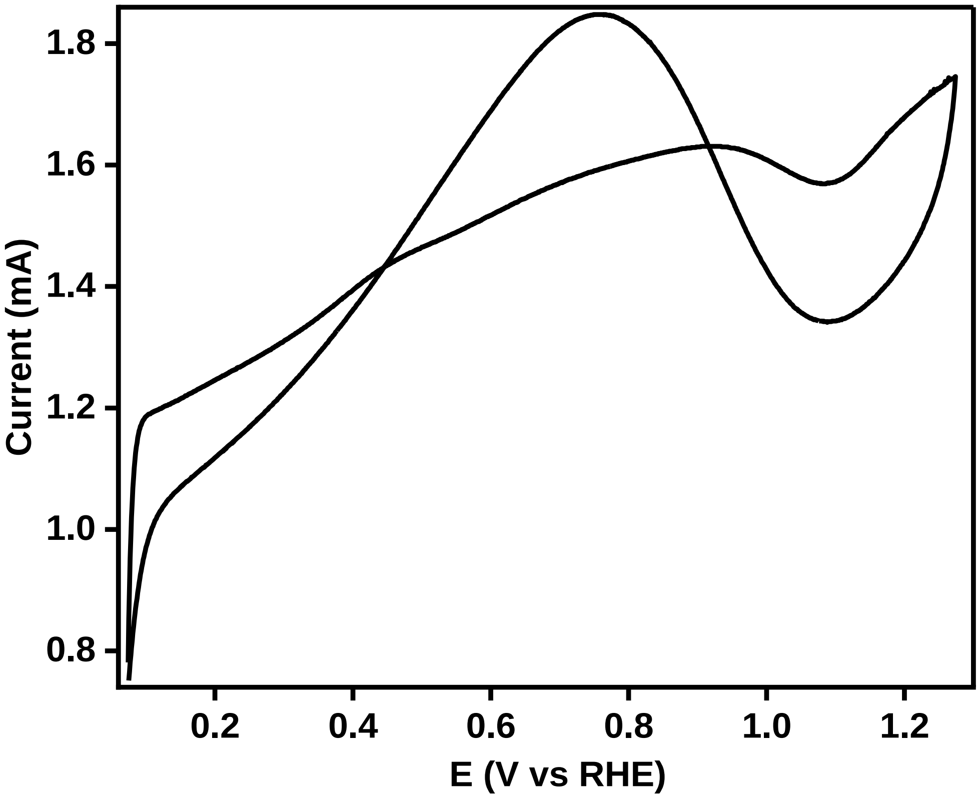

Figure S9. Cyclic voltammogram in 0.5 M H_2SO_4 using liquid Pt-Ga for ECSA measurement.

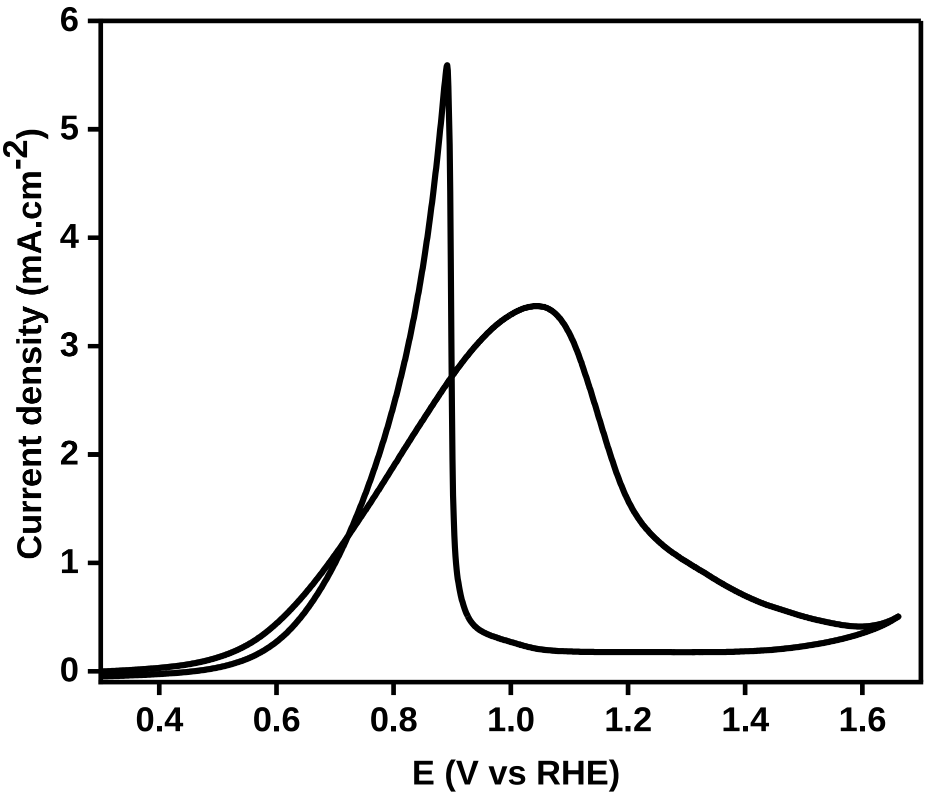

Figure S10. Electrode activity based on electrochemical surface area.

Table S1. Effect of KOH concentrations on peak potential and current (Without Additives).

KOH Concentration (M)	Peak Potential (V vs RHE)	Current (mA)
0.01	1.01	9.14
0.03	0.98	12.68
0.1	0.92	11.14
0.3	0.82	10.11
0.5	0.81	7.73
1.5	0.59	2.46
3.0	0.59	3.00

Table S2. Electrochemical activities of various Pt- based catalysts deployed for ethanol oxidation.

Catalyst type	Mass activity	Refs.
0.5% Pt-Ga	13.5 A· mg^{-1}_{Pt}	Present study
PtRh NWs/C	1.55 A· mg^{-1}_{Pt}	[3]
AuPtIr	58 A· mg^{-1}	[4]
Ultra-small Pt nanoparticles	5.22 A· mg^{-1}	[5]
Pt_3Ag alloy wavy nanowires	6.1 A· mg^{-1}	[6]
Pt_3Sn NPs/C	1.46 A· mg^{-1}	[7]
Pt Atomic Layers	1.166 A· $mg^{-1}{}_{Pt}$	[2]
PtIrNi nanocrystals	3.8 A· mg^{-1}	[8]
PtCoCu/CNTs composite	3.58 A· mg^{-1}_{Pt}	[9]
Hollow Structured Pt–Rh	2.488 A· mg^{-1}_{Pt}	[10]

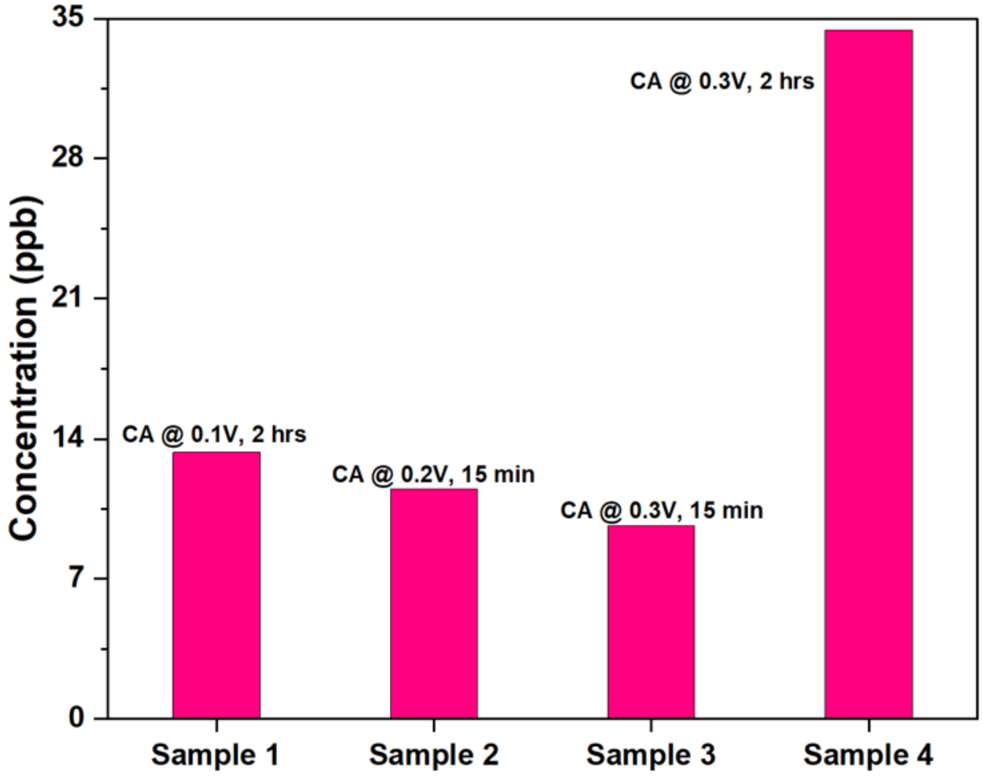

Figure S11. ICP-MS analysis for Ga from samples at different voltages and time intervals.

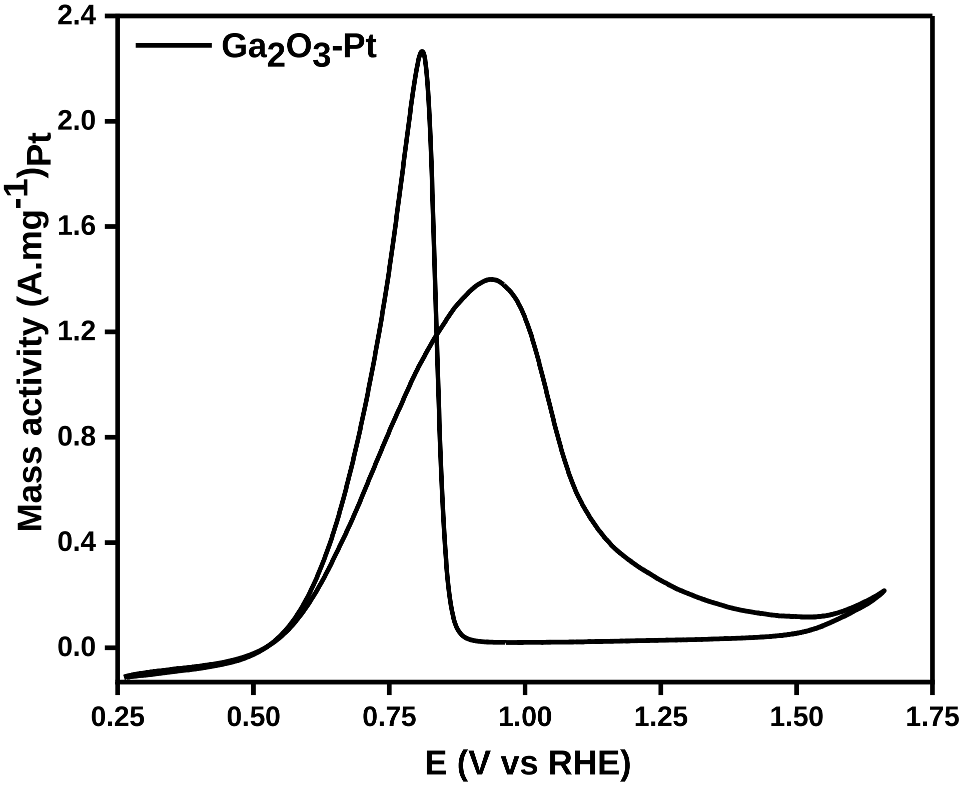

Figure S12. Cyclic voltammetry analysis of ethanol oxidation using Ga_2O_3-Pt catalyst.

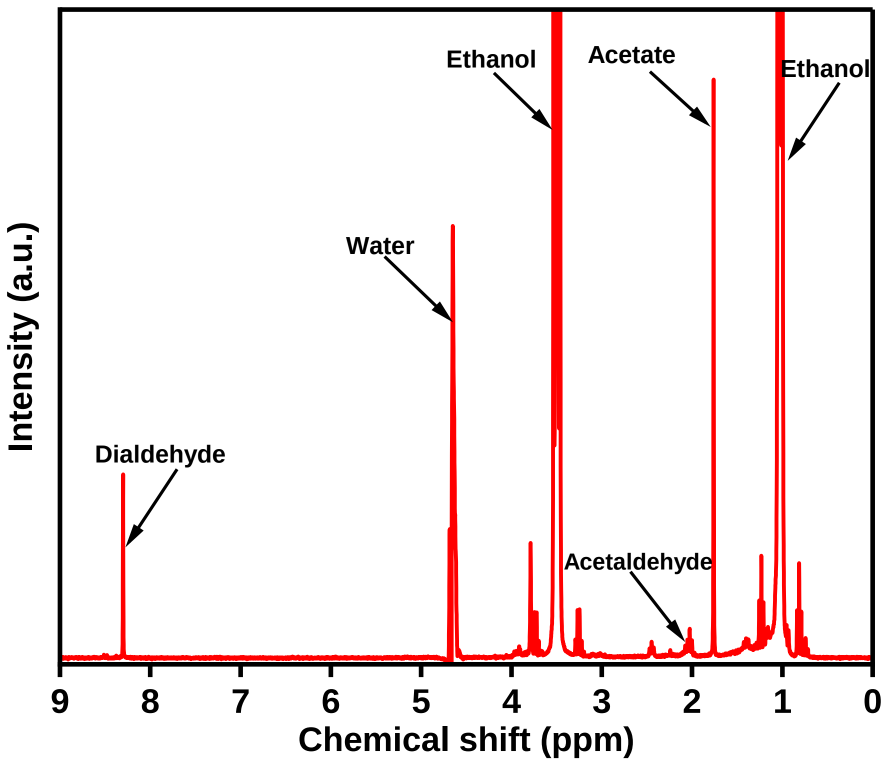

Figure S13. 1HNMR analysis of electrolyte sample for product detection after EOR.

References

[1] C. J. Parker, V. Krishnamurthi, K. Zuraiqi, C. K. Nguyen, M. Irfan, F. Jabbar, *et al.*, "Synthesis of Planet - Like Liquid Metal Nanodroplets with Promising Properties for Catalysis," *Advanced Functional Materials,* p. 2304248, 2023.

[2] Y. Chen, J. Pei, Z. Chen, A. Li, S. Ji, H. Rong, *et al.*, "Pt atomic layers with tensile strain and rich defects boost ethanol electrooxidation," *Nano Letters,* vol. 22, pp. 7563-7571, 2022.

[3] Y. Zhu, L. Bu, Q. Shao, and X. Huang, "Subnanometer PtRh nanowire with alleviated poisoning effect and enhanced C–C bond cleavage for ethanol oxidation electrocatalysis," *ACS Catal,* vol. 9, pp. 6607-6612, 2019.

[4] Z. Liang, L. Song, S. Deng, Y. Zhu, E. Stavitski, R. R. Adzic, *et al.*, "Direct 12-electron oxidation of ethanol on a ternary Au (core)-PtIr (shell) electrocatalyst," *J Am Chem Soc,* vol. 141, pp. 9629-9636, 2019.

[5] C. Hu, Y. Zhou, M. Xiao, and G. Yu, "Precise size and dominant-facet control of ultra-small Pt nanoparticles for efficient ethylene glycol, methanol and ethanol oxidation electrocatalysts," *Int J Hydrogen Energy,* vol. 45, pp. 4341-4354, 2020.

[6] X. Fu, C. Wan, A. Zhang, Z. Zhao, H. Huyan, X. Pan, *et al.*, "Pt 3 Ag alloy wavy nanowires as highly effective electrocatalysts for ethanol oxidation reaction," *Nano Res,* vol. 13, pp. 1472-1478, 2020.

[7] Y. Zhu, L. Bu, Q. Shao, and X. Huang, "Structurally ordered Pt3Sn nanofibers with highlighted antipoisoning property as efficient ethanol oxidation electrocatalysts," *ACS Catal,* vol. 10, pp. 3455-3461, 2020.

[8] Y. H. Ahmad, A. T. Mohamed, K. M. Youssef, S. Kundu, K. A. Mkhoyan, and S. Y. Al-Qaradawi, "Rational synthesis of ternary PtIrNi nanocrystals with enhanced poisoning tolerance for electrochemical ethanol oxidation," *Electrochem commun,* vol. 101, pp. 61-67, 2019.

[9] M. Nie, Z. Xu, Y. Wang, H. You, L. Luo, B. Li*, et al.*, "Ultrafast synthesis of efficient TS-PtCoCu/CNTs composite with high feed-to-product conversion rate by Joule heating for electrocatalytic oxidation of ethanol," *J Colloid Interface Sci,* vol. 660, pp. 334-344, 2024.
[10] K.-H. Kim, G. M. Hobold, K. J. Steinberg, and B. M. Gallant, "Confinement effects of hollow structured Pt–Rh electrocatalysts toward complete ethanol electrooxidation," *ACS Nano,* vol. 17, pp. 14176-14188, 2023.
